# The functional status of vestibular otolith and conductive pathway in patients with unilateral idiopathic sudden sensorineural hearing loss

**DOI:** 10.3389/fneur.2023.1237516

**Published:** 2023-07-20

**Authors:** Jiali Shen, Xiaobao Ma, Qing Zhang, Jianyong Chen, Lu Wang, Wei Wang, Kuan He, Jin Sun, Qin Zhang, Xiangping Chen, Maoli Duan, Yulian Jin, Jun Yang

**Affiliations:** ^1^Department of Otorhinolaryngology-Head and Neck Surgery, Xinhua Hospital, Shanghai Jiaotong University School of Medicine, Shanghai, China; ^2^ENT Department, Shanghai Jiaotong University School of Medicine Ear Institute, Shanghai, China; ^3^ENT Department, Shanghai Key Laboratory of Translational Medicine on Ear and Nose Diseases, Shanghai, China; ^4^Ear Nose and Throat Patient Area, Trauma and Reparative Medicine Theme, Karolinska University Hospital, Stockholm, Sweden; ^5^Division of Ear, Nose, and Throat Diseases, Department of Clinical Science, Intervention and Technology, Karolinska Institute, Stockholm, Sweden

**Keywords:** idiopathic sudden sensorineural hearing loss, vestibular evoked myogenic potential, hearing improvement, vestibular otolith, vestibular conductive pathway

## Abstract

**Background:**

The cause of idiopathic sudden sensorineural hearing loss (ISSNHL) remains unknown. It has been found that the functional status of the vestibular otolith is relevant to its prognosis; however, the evaluation of the vestibular otolith (intra-labyrinth) and superior and inferior vestibular nerve pathways (retro-labyrinth) in ISSNHL patients is not well-documented.

**Objective:**

This study aimed to investigate the functional status of the vestibular otolith and conductive pathway in patients with unilateral ISSNHL and analyze the correlations between vestibular evoked myogenic potentials (VEMPs) and hearing improvement after treatment.

**Methods:**

A total of 50 patients with unilateral ISSNHL underwent a battery of audio-vestibular evaluations, including pure tone audiometry, middle ear function, air-conducted sound-cervical VEMP (ACS-cVEMP), ACS-ocular VEMP (ACS-oVEMP), galvanic vestibular stimulation-cervical VEMP (GVS-cVEMP), and GVS-ocular VEMP (GVS-oVEMP). The results of auditory and VEMPs were retrospectively analyzed.

**Results:**

The abnormal rates of ACS-cVEMP, ACS-oVEMP, GVS-cVEMP, and GVS-oVEMP in affected ears were 30, 52, 8, and 16%, respectively. In affected ears, the abnormal rate of ACS-oVEMP was significantly higher than that of ACS-cVEMP (*p* = 0.025), while it was similar between GVS-cVEMP and GVS-oVEMP (*p* = 0.218). Compared with GVS-cVEMP, affected ears presented with a significantly higher abnormal rate of ACS-cVEMP (*p* = 0.005), and the abnormal rate of ACS-oVEMP was significantly higher than that of GVS-oVEMP (*p* < 0.001). No significant difference existed in latency and amplitude between affected and unaffected ears in ACS-VEMPs or GVS-VEMPs (*p* > 0.05). The abnormal rate of VEMPs in the poor recovery group was significantly higher than that of the good recovery group (*p* = 0.040). The abnormality percentages of ACS-oVEMP and GVS-oVEMP in the poor recovery group were significantly higher than that of the good recovery group (*p* = 0.004 and 0.039, respectively). The good hearing recovery rates were 76.47% in the normal VEMPs group, 58.33% in the intra-labyrinth lesion group, and 22.22% in the retro-labyrinth lesion group. Hearing recovery worsened as a greater number of abnormal VEMPs was presented.

**Conclusion:**

Besides Corti's organ, the impairment of otolithic organs was prominent in patients with ISSNHL. The normal VEMPs group had the highest rate of good recovery, followed by the intra-labyrinth lesion group and the retro-labyrinth lesion group presented with the lowest recovery rate. Abnormalities in ACS-oVEMP and/or GVS-oVEMP were indicators of a poor prognosis.

## 1. Introduction

Idiopathic sudden sensorineural hearing loss (ISSNHL) was defined as a sudden hearing loss that occurs within 72 h, and pure tone audiometry results show a decline at least in the three adjacent frequencies (>30 dBHL) without any identifiable cause (1), which may lead to difficulty in speech recognition and sound localization, especially in noisy environments, and has a significant negative impact on the quality of life and mental status of patients (2–4).

Although several etiologies of ISSNHL, such as viral infection, inflammation, inner ear circulation disorders, cochlear membrane breaks, and vascular occlusion, have been suggested, the exact pathogenesis is still unclear (3, 5). Due to the anatomical proximity of the cochlea and vestibule, ISSNHL is frequently accompanied by vestibular dysfunction (6, 7). Multiple studies have shown that patients with ISSSNHL also have clinical symptoms of dizziness, indicating that the vestibular organ may get involved. It was reported that nearly 40–55% of patients with ISSNHL suffered from vestibular dysfunction and were more commonly associated with severe hearing loss rather than mild and moderate hearing loss (8–13).

Considering that a high proportion of patients with ISSNHL have vestibular dysfunction, it is of great importance to evaluate vestibular function in these populations. The most commonly used tests include the caloric test, cervical vestibular evoked myogenic potential (cVEMP), and ocular VEMP (oVEMP), as well as video head impulse test (vHIT) (14–16). The caloric test can be conducted to assess lateral semicircular canal (LSCC) and superior vestibular nerve function. cVEMP can be used for investigating the function of the saccule and the inferior vestibular nerve, while oVEMP can be applied for evaluating the function of the utricle and the superior vestibular nerve. Researchers found that vestibular function can predict the hearing outcomes of patients with ISSNHL to a certain extent (8–10, 17, 18). A correlation was found between poor prognosis and vestibular dysfunction. However, most of them mainly applied air-conducted sound VEMPs (ACS-VEMPs) to evaluate the functional status of the vestibular otolith. There are few studies on the evaluation of the vestibular otolith (intra-labyrinth) and the superior and inferior vestibular nerve pathways (retro-labyrinth). In fact, ACS-VEMPs can only evaluate the integrity of the vestibular otolith conductive pathway; they are unable to distinguish intra-labyrinth or retro-labyrinth lesions (15, 19). Damage to anywhere in the conductive pathway can result in an abnormal ACS-cVEMP or oVEMP. However, since galvanic vestibular stimulation (GVS) directly stimulates vestibular afferents, it could be evoked in patients with only labyrinthine deficits (14, 20). Therefore, the combined application of ACS-VEMPs and GVS-VEMPs could be more efficient in localization diagnosis and may contribute to the prediction of prognosis (18–20). The purpose of this study was to investigate the functional status of the vestibular otolith and conductive pathway in patients with unilateral ISSNHL and analyze the relationship between VEMPs and hearing prognosis.

## 2. Materials and methods

### 2.1. Subjects

A retrospective study was performed on 50 patients with ISSNHL who were hospitalized at the Department of Otolaryngology-Head and Neck Surgery, Xinhua Hospital, affiliated with Shanghai Jiao Tong University School of Medicine from October 2019 to March 2023, including 26 male participants and 24 female participants aged between 8 and 75 years (an average of 45.87 ± 20.91 years). There were 28 ears with severe hearing loss and 22 ears with profound hearing loss. For the entire cohort, 28 patients had hearing loss in the left ear and 22 in the right ear, and 25 patients had vertigo. Patients were included if the results of VEMPs were normal on the contralateral unaffected ear, which could eliminate the influence of advanced age.

The inclusion criteria were as follows: (1) unilateral sudden sensorineural hearing loss without apparent cause at least in the adjacent three-frequency hearing loss of ≥30 dB HL in 72 h; (2) initiation of treatment within 20 days after onset; (3) underwent all the required tests; (4) Type A tympanogram in both ears; and (5) the same comprehensive treatment plan was used during hospitalization. The exclusion criteria were as follows: (1) abnormal results of VEMPs on the contralateral healthy ear; (2) external and middle ear diseases; (3) space-occupying lesions of the internal auditory canal and central organic pathology; and (4) sensorineural hearing loss due to noise exposure or ototoxic drugs.

### 2.2. Methods

#### 2.2.1. Audiological assessment

A tympanogram was obtained by the Interacoustics AT235H Middle Ear Analyzer (Interacoustics, Denmark). Type A at 226 Hz probe tone was considered a normal middle ear function.

Pure-tone audiometry was conducted in a soundproof room using an audiometer (Type Astera, Madsen, Denmark). The pure-tone average (PTA) is the average of the 0.5, 1, 2, and 4 kHz pure-tone thresholds. According to the latest standards of the World Health Organization, PTA <20 dB HL is defined as normal hearing. Repeated pure-tone audiometry was carried out before and after the 10-day treatment. The hearing outcome was classified as good recovery (referring to PTA gain ≥15 dB HL) and poor recovery (hearing improvement <15 dB HL). If no response was obtained for a certain frequency, which exceeded the maximum output of the audiometer (120 dB HL), 120 dB HL was used as the estimated hearing threshold.

#### 2.2.2. ACS-VEMPs

ACS-VEMPs were recorded by the electrophysiological device (Neuropack MEB-9400, NIHON KOHDEN, Japan). A sound stimulus of Tone-Burst 500 Hz (the rise/fall time = 1 ms and the plateau time = 2 ms) at 132 dB peSPL was presented monaurally through a calibrated headphone TDH-39 at a rate of 5 Hz. A minimum of 100 sweeps were averaged and at least repeated twice to verify the waveform repeatability. The electromyogram (EMG) signals were amplified and bandpass filtered between 10 and 3,000 Hz.

For ACS-cVEMP, the two recording electrodes were placed on the upper third of the bilateral sternocleidomastoid muscles (SCMs). The two reference electrodes were placed on the sternal end of the SCM. Then the ground electrode was placed in the middle of the forehead. Patients were asked to rotate their heads toward the shoulder in a sitting position, keeping the SCMs activated and tense until the stimulus sound stopped.

ACS-oVEMP was also performed in a sitting position. The two recording electrodes were placed 1 cm below the middle of the contralateral lower eyelid, the reference electrodes were placed below the same side of the recording electrodes, and the ground electrode was placed in the middle of the forehead. Patients were required to maintain eye gaze upward for 25–30° when hearing a single acoustic stimulus and minimize blinking to maintain tension in the inferior oblique muscle until the stimulation stopped.

#### 2.2.3. GVS-VEMPs

GVS-VEMPs were performed by the same device. The electrode placement of GVS-VEMPs was similar to that of ACS-VEMPs, but there was a set of cathode and anode electrodes for direct current stimulation. The cathode of direct current stimulation was placed at the mastoid, and the anode was placed over the forehead (21).

GVS-cVEMP: The initial stimulation intensity was 3.0 mA/1 ms (stimulation rate 5 Hz, bandpass filter 20–2,000 Hz, and 50 sweeps were averaged). The waveform of muscle relaxation was subtracted from the waveform of muscle contraction to eliminate the artifact of the mechanical wave and obtain the final waveform. The method of muscle contraction was the same as in ACS-cVEMP.

GVS-oVEMP: The initial stimulation intensity was 3.0 mA/1 ms (stimulation rate 5 Hz, bandpass filter 1–1,000 Hz, and 50 sweeps were averaged). The waveforms of the extraocular muscles were recorded during upward gaze (extraocular muscles contraction) and downward gaze (extraocular muscles relaxation), and the final GVS-oVEMPs waveform was obtained by subtracting the waveform of muscle relaxation from muscle contraction.

To verify the repeatability of the waveform, the process was repeated at least twice. If 3.0 mA cannot elicit repeatable waveforms, the stimulation intensity can be appropriately increased according to the patient's tolerance level but usually does not exceed 5.0 mA. Characteristics of latencies, amplitudes, and the interaural asymmetry ratio (IAR) were recorded. IAR = (AL-AS)/(AL+AS) × 100%, where AL is the larger corrected amplitude and AS is the smaller corrected amplitude (22, 24, 26). Absent response, latency exceeding the normal limit, or IAR >30% was considered abnormal in our laboratory.

### 2.3. Statistical analyses

All statistical analyses were performed using SPSS 26 (SPSS Inc., Chicago, IL, United States). A chi-square test was used to evaluate the demographics of the two groups of ISSNHL patients and the abnormal rates of various VEMPs. The pre-treatment and post-treatment PTAs were compared using the paired *t*-test. Latencies and amplitudes of various modes of VEMPs were determined by independent *t*-test for parametric variables and Mann-Whitney *U*-test for non-parametric variables. A chi-square test with Bonferroni correction was applied to evaluate the good hearing recovery rate among different groups. Significance was determined at *p* < 0.05.

## 3. Results

### 3.1. Subject characteristics

**Figure 1:**
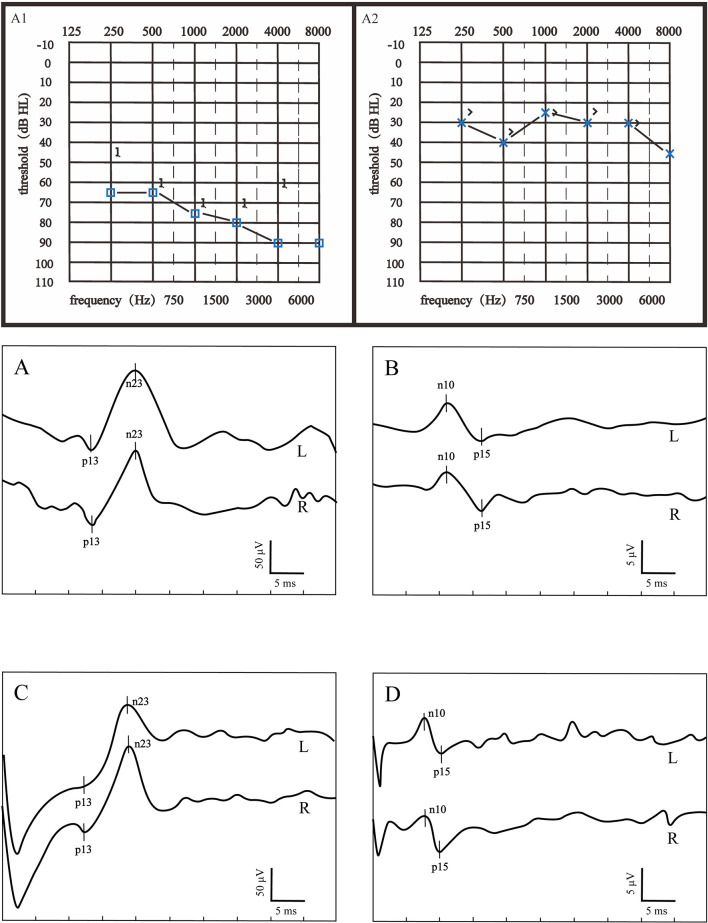
Audiograms and VEMPs of a patient with good hearing recovery. **(A1)** showed the initial hearing and **(A2)** showed the after-treatment hearing. **(A)** ACS-cVEMP; **(B)** ACS-oVEMP; **(C)** GVS-cVEMP; **(D)** GVS-oVEMP; L, left ear; R, Right ear. The left ear was the affected ear. There was no significant difference in waveform between the healthy and affected ears in **(A–D)**.

**Figure 2:**
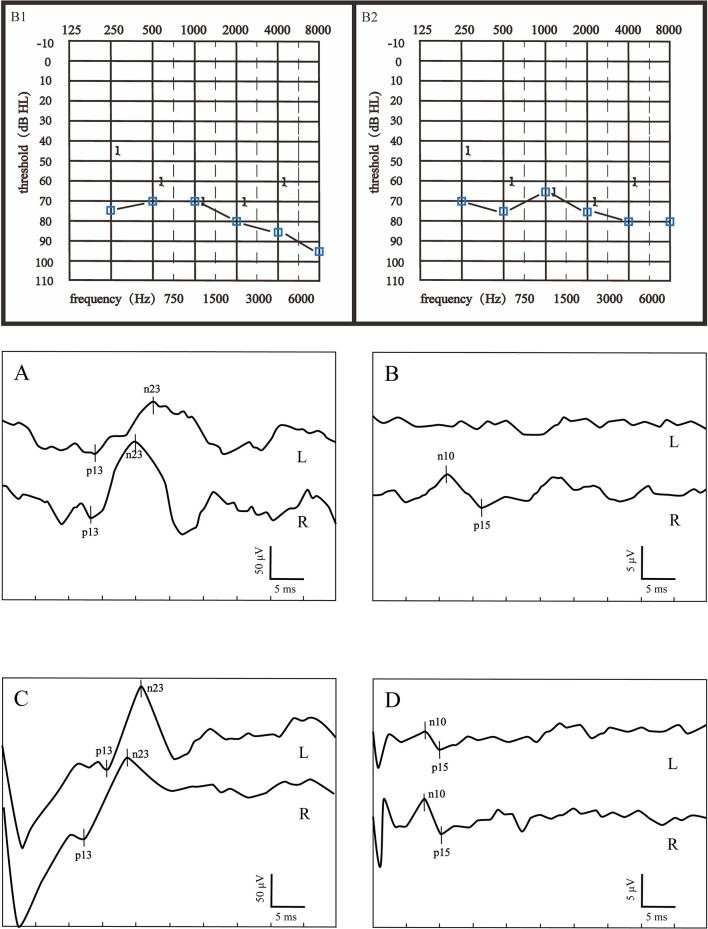
Audiograms and VEMPs of a patient with poor hearing recovery. **(B1)** showed the initial hearing and **(B2)** showed the audiogram after treatment. **(A)** ACS-cVEMP; **(B)** ACS-oVEMP; **(C)** GVS-cVEMP; **(D)** GVS-oVEMP; L, left ear; R, Right ear. The left ear was the affected ear. This patient presented with longer p13 and n23 latencies both in ACS-cVEMP and GVS-cVEMP, an absent waveform in ACS-oVEMP, and amplitude asymmetry in GVS-oVEMP.

According to the inclusion criteria, 50 patients with severe to profound ISSNHL were enrolled in this study. Based on the hearing recovery, they were categorized into two groups, namely, the good recovery (GR) group and the poor recovery (PR) group, with 29 cases (58%) in the GR group and 21 cases (42%) in the PR group. Figure 1 displays audiograms and VEMPs of a patient with good hearing recovery. Figure 2 depicts the initial hearing, after-treatment hearing, and VEMPs of a patient with poor hearing recovery. Demographics and results of chi-square and Mann-Whitney *U*-tests in the two groups are shown in Table 1. Our results showed there was no significant difference in gender, affected side, age, or initial hearing loss between the two groups (*p* > 0.05). However, the number of patients accompanied by vestibular symptoms and the presence of abnormal VEMPs were significantly higher in the PR group (*p* = 0.010 and 0.040, respectively).

**Table 1:** Clinical characteristics of patients in good and poor recovery groups.

**Variables**	**GR group (*n* = 29)**	**PR group (*n* = 21)**	***p*-value**
Gender (male:female)	14:15	12:9	0.536
Affected side (left:right)	16:13	12:9	0.890
VEMPs (normal:abnormal)	13:16	4:17	0.040^*^
Age (years)	34.59 ± 19.95	43.29 ± 23.32	0.215
Initial hearing loss (dB)	88.97 ± 22.70	82.38 ± 33.01	0.637
Vestibular symptoms, *n* (%)	10/29 (34.48%)	15/21 (71.42%)	0.010^*^

n, number of ears.

* *p* < 0.05.

### 3.2. Abnormal rate of VEMPs in affected ears

As shown in Table 2, in the 50 affected ears, the abnormal rates of ACS-cVEMP, ACS-oVEMP, GVS-cVEMP, and GVS-oVEMP were 30, 52, 8, and 16%, respectively. Specifically, there were eight absent responses, three delayed responses, and four smaller amplitude responses in ACS-cVEMP. In total, 23 absent responses and 3 asymmetric responses were observed in ACS-oVEMP. For GVS-cVEMP, only one absent response and three asymmetric responses were noted. Meanwhile, four absent responses, two delayed responses, and two amplitude reduction responses were discovered in GVS-oVEMP. The abnormal rate of ACS-cVEMP was significantly higher than that of GVS-cVEMP (*p* = 0.005). Similar to the results of cVEMP, the abnormal rate of ACS-oVEMP significantly exceeded that of GVS-oVEMP in affected ears (*p* < 0001). No significant difference existed between the abnormal rates of GVS-cVEMP and GVS-oVEMP (*p* = 0.218). However, the abnormal rate of ACS-oVEMP was significantly higher than that of ACS-cVEMP (*p* = 0.025).

**Table 2:** Comparison of abnormal rates of VEMPs in affected ears.

	**ACS-oVEMP (26/50, 52%)**	**GVS-cVEMP (4/50, 8%)**
ACS-cVEMP (15/50, 30%)	*p* = 0.025^*^	*p* = 0.005^*^
GVS-oVEMP (8/50, 16%)	*p* < 0.001^*^	*p* = 0.218

* *p* < 0.05.

### 3.3. Comparison of latency and amplitude between the affected and unaffected ears

The descriptive data, including the mean and standard deviation (SD) of the latency and amplitude of VEMPs in affected and unaffected ears, were displayed in Tables 3, 4. The results indicated that there was no significant difference in these parameters between affected and unaffected ears in ACS-cVEMP, ACS-oVEMP, GVS-cVEMP, or GVS-oVEMP (*p* > 0.05).

**Table 3:** Comparison of latency and amplitude of ACS-VEMPs between the affected and unaffected ears.

**Group**	***n***	**ACS-cVEMP**			**ACS-oVEMP**		
		**p13 latency (ms)**	**n23 latency (ms)**	**Amplitude (μV)**	**n10 latency (ms)**	**p15 latency (ms)**	**Amplitude (μV)**
Affected ears	50	16.80 ± 2.22	24.61 ± 2.85	214.20 ± 143.90	11.17 ± 0.92	15.32 ± 1.32	3.57 ± 2.03
Unaffected ears	50	16.44 ± 1.91	23.89 ± 2.72	260.34 ± 156.85	11.2 ± 0.86	15.59 ± 1.32	4.62 ± 3.22
*p*-value		0.243	0.056	0.058	0.715	0.429	0.100

**Table 4:** Comparison of latency and amplitude of GVS-VEMPs between the affected and unaffected ears.

**Group**	***n***	**GVS-cVEMP**			**GVS-oVEMP**		
		**p13 latency (ms)**	**n23 latency (ms)**	**Amplitude (μV)**	**n10 latency (ms)**	**p15 latency (ms)**	**Amplitude (μV)**
Affected ears	50	12.03 ± 1.66	20.18 ± 2.09	157.43 ± 95.14	8.10 ± 0.83	11.45 ± 1.11	6.76 ± 5.02
Unaffected ears	50	12.26 ± 2.20	19.82 ± 2.22	150.16 ± 76.58	8.20 ± 0.77	11.58 ± 1.31	6.43 ± 4.43
*p*-value		0.404	0.172	0.800	0.385	0.691	0.627

*n*, number of ears.

### 3.4. Relationship between hearing outcomes and VEMP results

**Figure 3:**
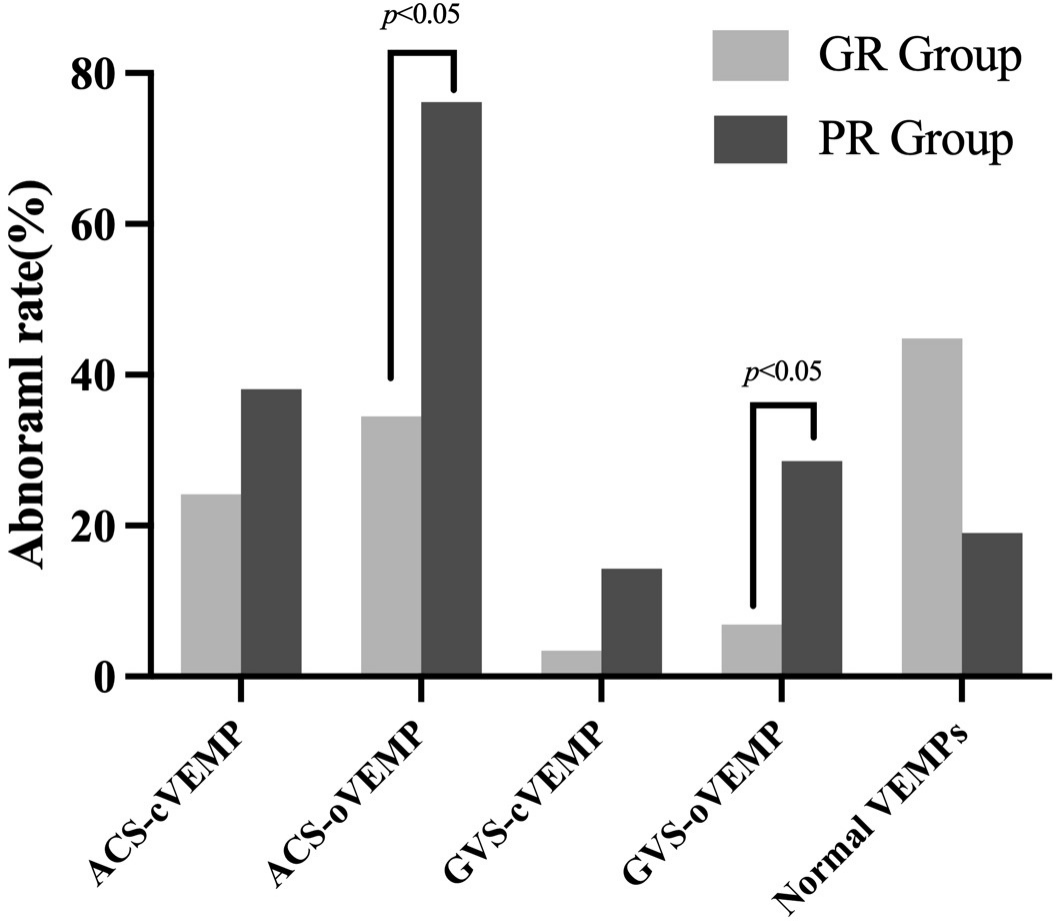
Abnormal rate of VEMPs in good and poor recovery groups. The light gray box plot represented a good recovery group (*n* = 29 ears); the dark gray box plot represented a poor recovery group (*n* = 21 ears). The abnormal rates of ACS-oVEMP and GVS-oVEMP were significantly higher in the PR group than in the GR group (*p* = 0.004, 0.039, respectively). No significant difference was found in ACS-cVEMP, GVS-cVEMP, or normal VEMPs (*p* = 0.288, 0.163, and 0.058, respectively).

According to the VEMP results, vestibular dysfunction locations were categorized into intra-labyrinth and retro-labyrinth lesions. The normal VEMPs group refers to normal results in ACS-VEMPs and GVS-VEMPs. Intra-labyrinth lesion refers to abnormal ACS-cVEMP and/or ACS-oVEMP but normal GVS-cVEMP and GVS-oVEMP. Retro-labyrinth lesion refers to abnormal GVS-cVEMP and/or GVS-oVEMP. As shown in Figure 3, abnormal rates of ACS-oVEMP and GVS-oVEMP were significantly higher in the PR group than in the GR group (*p* = 0.004 and 0.039, respectively). However, no significant difference was observed in terms of abnormality percentage in ACS-cVEMP, GVS-cVEMP, or normal VEMPs between the GR and PR groups (*p* = 0.288, 0.163, and 0.058, respectively). Additionally, 24 affected ears suffered from intra-labyrinth lesion, including three with saccule dysfunction, 13 with utricle dysfunction, and eight with abnormal function both in the saccule and utricle; nine affected ears showed lesions in retro-labyrinth; and 17 affected ears had normal function in the vestibular otolith and conductive pathway. The good hearing recovery rates are shown in Table 5. The normal VEMPs group had the highest rate of good recovery, followed by the intra-labyrinth lesion group, and the retro-labyrinth lesion group presented with the lowest recovery rate. Table 6 displays the good recovery rate in patients with different numbers of abnormal VEMPs. The rates were 76.47% in patients with four normal VEMPs, 61.90% in patients with one abnormal VEMP, 33.33% in patients with two abnormal VEMPs, and 16.67% in patients with three or four abnormal VEMPs, suggesting that the hearing recovery worsened as a greater number of abnormal VEMPs presented.

**Table 5:** Comparison of a good recovery rate with different VEMP results.

	***n***	**Good recovery rate**	***p*-value**
Intra-labyrinth	24	14/24 (58.33%)	0.029^*^
Retro-labyrinth	9	2/9 (22.22%)	
Normal VEMPs	17	13/17 (76.47%)	

*n*, number of ears.

* *p* < 0.05.

**Table 6:** Good recovery rate in patients with different numbers of abnormal VEMPs.

	***n***	**Good recovery rate**	***p*-value**
Normal VEMPs	17	13/17 (76.47%)	< 0.01^*^
One abnormal VEMP	21	13/21 (61.90%)	
Two abnormal VEMPs	6	2/6 (33.33%)	
Three or four abnormal VEMPs	6	1/6 (16.67%)	

*n*, number of ears.

* *p* < 0.05.

## 4. Discussion

According to the results, the rate of abnormalities in ACS-VEMPs was greater than in GVS-cVEMPs, suggesting that the otolith organs may be involved more frequently than vestibular afferents, which was consistent with previous studies (18, 22–25). Chang et al. (25) reported that the abnormal rate of ACS-cVEMP in patients with ISSNHL was significantly higher than that of GVS-cVEMP (60 vs. 37%) in affected ears. The abnormal rate of bone conducted vibration oVEMP (BCV-oVEMP) significantly exceeded that of GVS-oVEMP in affected ears (47 vs. 20%). Iwasaki et al. (18) found that all ISSNHL patients in their study presented with normal GVS-VEMPs, which implied that the lesion site of ISSNHL was within the labyrinth. Additionally, we found that the abnormal rate of ACS-oVEMP was significantly higher than that of ACS-cVEMP. Nevertheless, the abnormal rates of GVS-cVEMP and GVS-oVEMP were comparable. These findings suggested that the utricle was most susceptible to damage in patients with ISSNHL, followed by the saccule and vestibular nerve. Vestibular organs could be affected individually or simultaneously.

A recent meta-analysis reported that the utricle was the most easily affected organ in ISSNHL (7), which was in agreement with our results. Lim et al. (26) demonstrated the association between vestibular function and prognosis in 264 SSNHL patients and reported that the functions of vestibular organs, particularly the utricle and lateral semicircular canal, are associated with disease severity and hearing outcome. Wang et al. (27) conducted a retrospective study to evaluate the association between hearing characteristics/prognosis and the patterns of vestibular/cochlear lesions in SSNHL patients with vertigo and found that more cases of vestibular dysfunction appeared in the lateral semicircular canal and the utricle than in the saccule. Liu et al. (6) also reported that the abnormal rate of oVEMP was the highest, indicating that the utricle might be more prone to damage than the saccule. This could be explained by the differential effects of ischemia on the anterior and posterior vestibular arteries. It has been found that the pattern of vestibular organ dysfunction correlates with the blood supply pattern of the cochlea and vestibule (28, 29). cVEMP reflects saccular function, and perfusion to the saccule is mainly supplied by the posterior vestibular arteries, while oVEMP reflects the function of the utricle, and perfusion is mainly provided by the anterior vestibular artery. Considering that the vestibulocochlear and posterior vestibular arteries have more intraosseous collaterals than the anterior vestibular arteries, the saccule may be more resistant to ischemic damage due to the better collateral blood supply (26, 29). In addition, although the cochlea and saccule are primarily supplied by branches of the common cochlea artery, the deterioration of saccular function may be less severe than that of the cochlea in common cochlea artery infarction (26).

However, some researchers have reported inconsistent results. It has been found that vestibular dysfunction in patients with ISSNHL affects the vestibular organs close to the cochlea first (30). Atrophic changes in the saccule were observed in patients with SSNHL (16). Fujimoto et al. (30) classified SSNHL patients with vertigo based on vestibular dysfunction patterns and discovered that the cochlea was most susceptible to damage, followed by the cochlea and saccule and the cochlea-saccule-utricle-semicircular canal type. They attributed this phenomenon to the anatomy of the saccule and its proximity to the cochlea. Meanwhile, Chang et al. (31) reported that there was no statistically significant difference between the rates of abnormal cVEMP and oVEMP, no matter what kind of stimulus modes were used. The inconsistency might be related to the following reasons: first, the different characteristics of participants. Due to the close relationship between the cochlea and vestibule, the degree of hearing loss had an effect on the percentage of abnormalities. In addition, the response rate of VEMP was strongly correlated with age. When the age exceeds 60 years, the response rate may decrease (23). All unaffected ears in our study presented with normal VEMPs, which could exclude the influence of age on the results. Second, test conditions are different. The stimulus modality (air conducted, bone vibration, or galvanic stimulation), intensity, and test position (supine or sitting) are all related to the VEMP results. Furthermore, unequal diagnostic criteria in different institutions also lead to various interpretations.

It has been documented that VEMP test results have predictive value for hearing outcomes in SSNHL patients. Several recent reports have included the VEMP test in the evaluation of patients with ISSNHL, for whom abnormal results of vestibular examinations were associated with poor hearing recovery (3, 10, 24, 32, 33). Wang et al. (17) proposed that profound hearing loss with normal VEMP was associated with favorable hearing results. Liang et al. (12) found that patients with abnormal oVEMP or/and cVEMP had poor hearing outcomes, suggesting that oVEMP and cVEMP may be effective indicators for predicting the prognosis.

The improvement of the pure tone threshold is currently a common and international outcome index (34, 35). We classified all patients into good recovery or poor recovery groups according to the hearing improvement of the affected ear. Our results showed that the abnormal rate of VEMP in the PR group was significantly higher than that in the GR group. The abnormality percentage of ACS-oVEMP and GVS-oVEMP in the poor recovery group was significantly higher than that in the good recovery group (Figure 3), while no significant difference was observed in terms of ACS-cVEMP, GVS-cVEMP, or normal VEMPs, indicating that the oVEMP pathway was more commonly affected, which agreed with previous findings (8, 16, 36, 37). It has been noted that the superior division of the vestibular nerve was preferentially affected. The lateral bony channel of the superior vestibular nerve is seven times longer than the inferior vestibular and more than three times longer than the singular channel. Additionally, the superior vestibular nerve and arteriole travel through a relatively narrower passage compared with the inferior or singular nerves. From an anatomical perspective, this makes the superior vestibular nerve more susceptible to entrapment and possible ischemic labyrinthine changes (37, 38). In addition, we found ISSNHL patients with normal VEMPs had the highest good recovery rate, followed by the intra-labyrinth lesion group and the retro-labyrinth lesion group presented with the lowest recovery rate, which was consistent with the results of previous research (5, 25). GVS-VEMP would stimulate the most distal portion of the vestibular nerve (20); an abnormal GVS-VEMP result indicates that the lesion area has extended to the nerve. It has been reported that the degree of inner ear lesions is negatively correlated with the possibility of hearing recovery; the involvement of otoliths and/or vestibular nerves implies a wider range of diseases, indicating a poorer prognosis (17, 18, 39). In other words, vestibular nerve injury may be a discriminant indicator of severe disease and negatively correlated with hearing recovery. Furthermore, it was reported that abnormalities in GVS-cVEMP and/or GVS-oVEMP may indicate degenerative changes in the vestibular nerve; patients with a longer onset of disease are more likely to experience auditory and vestibular nerve dysfunction. The functional recovery of nerves may take a long time, thus affecting hearing recovery (31). However, Iwasaki et al. (18) found that all ISSNHL patients in their study presented with normal GVS-VEMPs and concluded that GVS-VEMPs were not related to recovery.

The cause of this discrepancy may possibly relate to the following factors. First, the degree of hearing loss, accompanying symptoms (vertigo and tinnitus), delays after the onset of hearing loss, and etiology can lead to a significant risk of selection bias and unmatched groups. The extent of vestibular abnormalities correlated well with the degree of hearing loss (24, 32). Nearly 50% of ISSNHL patients in the study by Iwasaki et al. (18) had mild to moderate initial hearing loss, while our study focused on severe to profound hearing loss. Although several etiologies have been suggested, the exact pathogen is still unclear. Vascular dysfunction and viral infection are considered to be the most common causes of ISSNHL. It was reported that vascular damage could increase blood viscosity, make the blood in a hypercoagulable state, cause microcirculation disorders in the inner ear, and lead to damage to cochlear hair cells, and ultrastructural changes (5). Viruses, such as the herpes simplex virus, varicella-zoster virus, mumps, cytomegalovirus, and rubella, have been considered to correlate with the pathogenesis of ISSNHL. The role of viral infection is unknown, but it may cause endolymphatic biochemical changes or intravascular coagulation, affecting hair cell function and further leading to neurodegenerative changes (18, 40, 41). Additionally, the criteria for hearing recovery, PTA calculation, hearing loss classifications, and vestibular function evaluation were different.

### 4.1. Limitations and future direction

Although some valuable results were achieved, there were still some limitations in our study. First, we focused on a subgroup of ISSNHL patients with severe to profound hearing loss, so the effect of the degree of hearing loss was not mentioned in this study, which should be further studied on a large sample scale. Second, VEMPs do not reflect canal function; a comprehensive evaluation is required in combination with other vestibular tests. Due to the insufficient sample size of this study, it was not grouped according to etiology, medical diseases, etc. The effect of these potential factors on hearing prediction should be incorporated into statistical analysis in future studies with larger sample sizes. In addition, we could also follow up on hearing and vestibular recovery at 1 and 3 months after treatment, making the study more comprehensive.

## 5. Conclusion

There were significant differences in the recovery of hearing loss in patients with ISSNHL. Patients with abnormal VEMPs have unfavorable hearing outcomes compared with those with normal VEMPs. Otolith organs are involved more frequently than afferents in patients with ISSNHL. Furthermore, the utricle was more susceptible compared to the saccule. The combination of ACS and GVS-VEMPs can better evaluate the lesion site and contribute to the clinical diagnosis, treatment, and prognosis evaluation of ISSNHL.

## Data availability statement

The original contributions presented in the study are included in the article/supplementary material, further inquiries can be directed to the corresponding authors.

## Ethics statement

This study was approved by the Ethics Committee of Xinhua Hospital Affiliated to Shanghai Jiaotong University School of Medicine (Approval No. XHEC-D-2022-259). Written informed consent to participate in this study was provided by the participants' legal guardian/next of kin.

## Author contributions

JSh was responsible for data interpretation and manuscript preparation. XM contributed to the data analysis. QingZ helped optimize the VEMP test procedure. JC contributed to the clinical consultation. LW, WW, KH, JSu, and QinZ were responsible for auditory tests and VEMP tests. XC contributed to the statistical consultation. MD and YJ reviewed and revised the manuscript and study design. JY was responsible for the research design and manuscript revision. All authors contributed to the article and approved the submitted version.
